# Supplementary material for: The Low-Temperature Photocurrent Spectrum of Monolayer MoSe2: Excitonic Features and Gate Voltage Dependence
Source: Nanomaterials (Basel). 2022 Jan 19;12(3):322. doi: 10.3390/nano12030322 (PMC8838275; doi:10.3390/nano12030322)
Supplement: Supplementary file 1 [file nanomaterials-12-00322-s001.zip › nanomaterials-1548102-supplementary.pdf]

Supplementary information to:

The low-temperature photocurrent spectrum of monolayer $MoSe_2$: Excitonic features and gate voltage dependence.

*Daniel Vaquero-Monte [1], Juan Salvador-Sánchez[1], Vito Clericò[1], Enrique Diez [1] and Jorge Quereda [1,2,]**

[1] Nanotechnology Group, USAL–Nanolab, Universidad de Salamanca, E-37008 Salamanca, Spain.

[2] GISC, Departamento de Física de Materiales, Universidad Complutense, E-28040 Madrid, Spain.

* e-mail: j.quereda@usal.es, jorquere@ucm.es

Table of contents

Supplementary Section S1. Raman characterization

Supplementary Section S2. Evaluation of the photodoping effect

Supplementary Section S3. Blue shift of neutral exciton

Supplementary Section S4. Fermi energy as a function of the gate voltage

S1. Raman Characterization

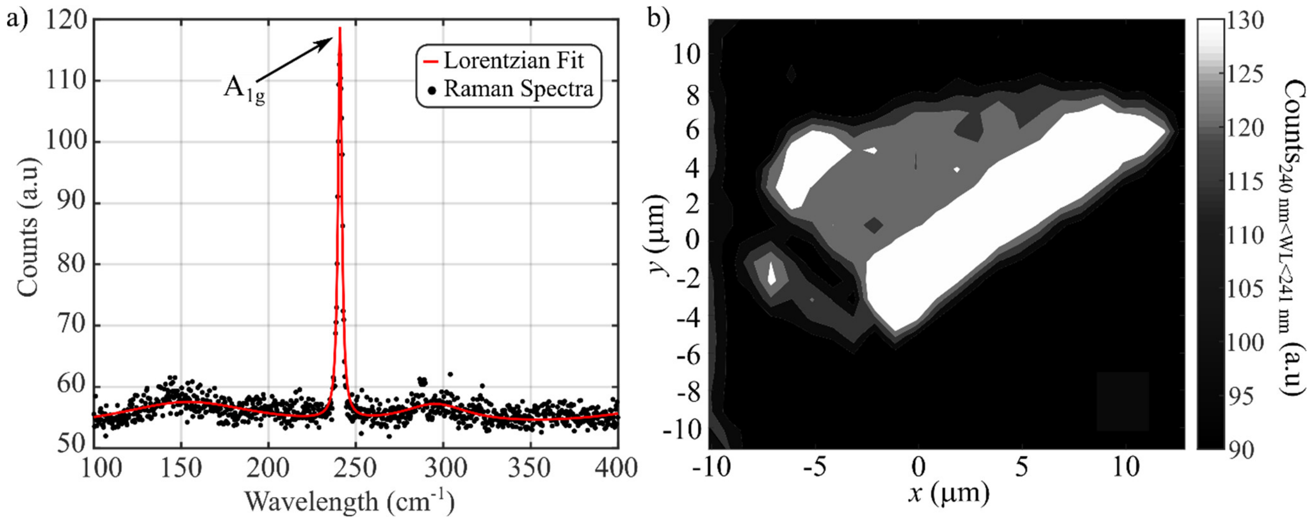

Figure S1. Raman characterization of the $MoSe_2$ thickness. a) Raman spectrum of the 1L-$MoSe_2$ flake used for the device of the main text (black dots) and lorentzian fit experimental data (red line). Raman peak A_{1g} is labelled in the figure. b) False color Raman map of the mean value of the counts in the raman spectra between wavelengths of 240 nm and 241 nm.

We determine the thickness of the $MoSe_2$ flake used for the device fabrication (figure 1 of the main text) using Raman spectroscopy. Figure S1a shows the Raman spectrum of the 1L-$MoSe_2$ flake. We fit the spectra to a multi-Lorentzian function. In the spectrum we observe a prominent peak at 240.82 cm^{-1}, which corresponds with the A_{1g} peak[1]. As the thickness of the $MoSe_2$ increases the A_{1g} peak shifts to higher wavelengths. Figure S1b shows a false colour map of the mean value of the counts between 240 and 241 cm^{-1} in the Raman spectra measured in different positions of the $MoSe_2$ flake. Colour scale used is depicted next to the Raman mapping. The different thickness of the flake can be identified clearly in the Figure. The white′colored area corresponds to monolayer $MoSe_2$.

S2. Evaluation of the photodoping effect

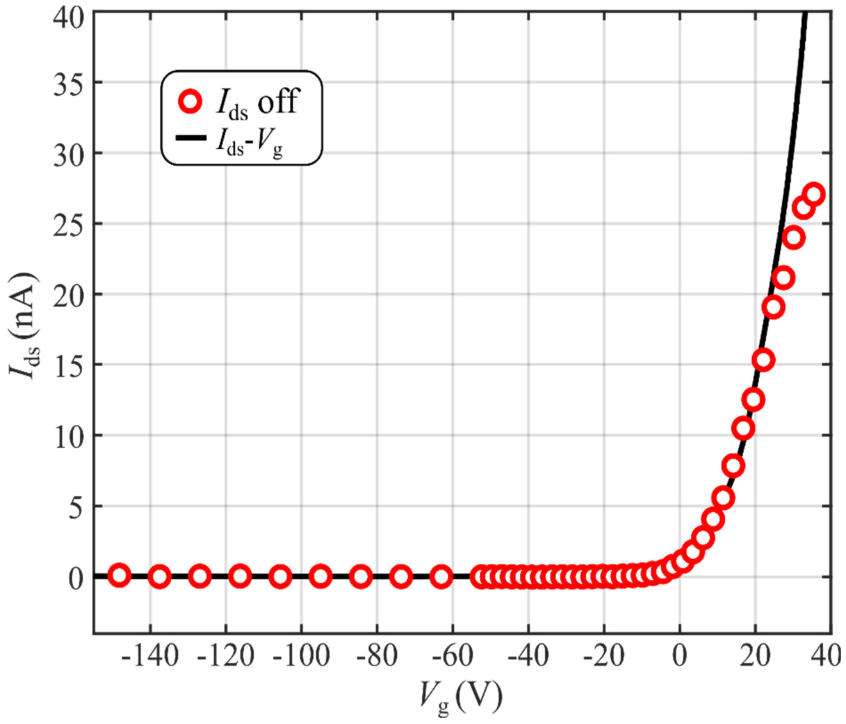

Figure S2. Comparison between the transfer curve of the device (black solid line) and I_{ds} of the device before the exposure of the device to the light source to perform the photocurrent spectroscopy measurements (red and white dots).

Photodoping effects are common in 2D phototransistors encapsulated in h-BN[2], and can cause slow drifts in the carrier density over time.

In order to rule out photogating effects affecting our gate-dependent photocurrent spectroscopy measurements, we measured a gate transfer curve of the device before exposure to light. Then, immediately before each spectral acquisition we measure the off current and compare it to the expected value for the applied gate voltage

Figure S2 shows the comparison between the transfer curve and the values of I_{ds} measured before acquiring the different photocurrent spectra, confirming that photodoping effects where negligible during the experiment.

S3. Blue shift of neutral exciton

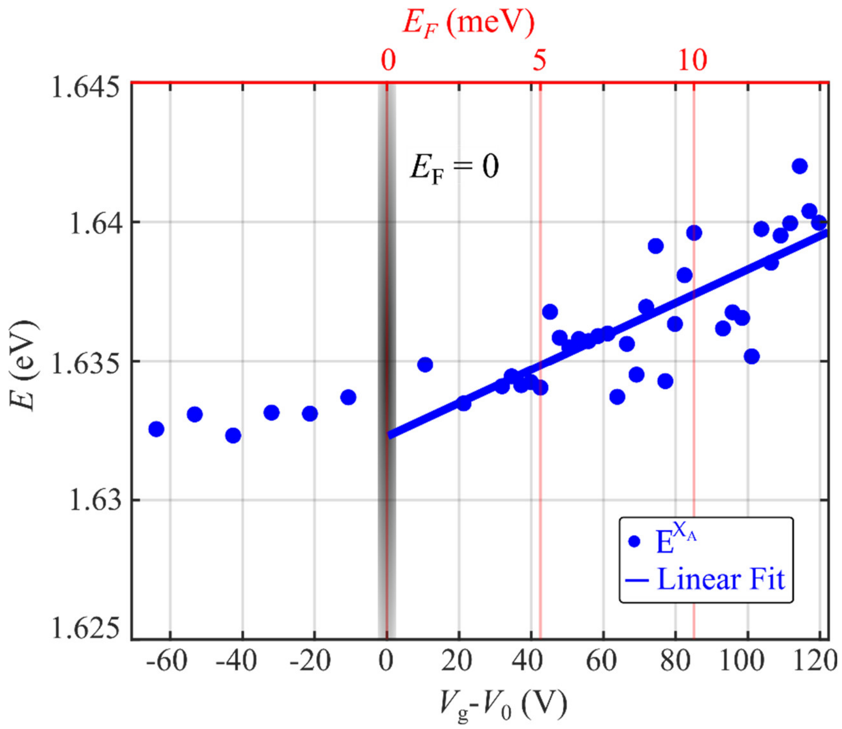

Figure S3. Blue-shift of exciton energy. Dependence on gate voltage and fermi energy of the exciton A transition energies.

Figure S3 shows the extracted peak energies of the neutral exciton A (X^{A}_{1s}) as a function of the gate voltage. We observe a clear blue shift for gate voltages over the charge neutrality point of the device. We use a linear relation to quantify the observed behaviour. The corresponding slope linear fit is 60.1 ± 8.5 meV/V, obtaining values comparable with similar studies in the previous literature[3]. We attribute this effect to the influence of the free charge carriers in the photocurrent spectra through many-body interactions[4].

S4. Fermi energy as a function of the gate voltage.

To estimate the Fermi energy shift caused by the gate voltage, we use the Fermi-Dirac statistics approximating $MoSe_2$ to a two-dimensional electron gas. The charge density in the device is equal to the density of occupied states in the conduction band, $\Omega(E_\mathrm{F})$.

$$\Omega(E_\mathrm{F}) = n = \int_{-\infty}^{E_F} g_{2\mathrm{D}}(E)\, f(E,\mu)\, dE \tag{S1}$$

where, $f(E,\mu)$ is the Fermi-Dirac distribution at 5 K and $g_{2\mathrm{D}}(E)$ is the density of states of a two-dimensional free electron Fermi gas, given by

$$g_{2\mathrm{D}}(E) = \frac{m_{eff}e}{2\pi\hbar^2} g_\mathrm{s} g_\mathrm{v} \tag{S2}$$

where m_{eff} is the effective mass of the electron in the conduction band ($m_{eff} = 0.8m_0$)[5], $g_\mathrm{s} = 2$ is the spin degeneracy, $g_\mathrm{v} = 2$ is the valley degeneracy and e is the electron charge. We use the charge neutrality point of the device, denoted in the main text as V_0, as the origin of energies. Approximating the fermi-dirac distribution at 5 K to the 0 K one, equation S1 yields,

$$n = \frac{2m_e^*}{\pi\hbar^2} E_F \tag{S3}$$

thus, the relation between the charge carriers density and the fermi energy, can be written as

$$\Delta n = \frac{2m_e^*}{\pi\hbar^2} E_\mathrm{F} \tag{S4}$$

We can now estimate Δn from a plane parallel capacitor model, where the SiO2 and the h-BN behaves as the dielectric layer of capacitor. Density of charge charrier using this model corresponds with

$$\Delta n = \frac{C_\mathrm{ox}}{e}\left(V_\mathrm{g} - V_0\right) \tag{S5}$$

where $\left(V_\mathrm{g} - V_0\right)$ is the variation of the gate voltage from the charge neutrality point of the device and C_ox is the capacitance of the SiO_2 and the bottom h-BN of the device. For this device we obtain a capacitance of $C_\mathrm{ox} = 12.53\ \mathrm{nF/cm^2}$.

Finally comparing the right-hand terms of equations S4 and S5 we obtain,

$$E_\mathrm{F} = \frac{C_{ox}\pi\hbar^2}{2m_\mathrm{e}^* e}\left(V_\mathrm{g} - V_0\right) \tag{S6}$$

For this device, we get $E_F/(V_g - V_0) = 0.117$ meV V[-1].

References

(1) Tonndorf, P.; Schmidt, R.; Böttger, P.; Zhang, X.; Börner, J.; Liebig, A.; Albrecht, M.; Kloc, C.; Gordan, O.; Zahn, D. R. T.; Michaelis de Vasconcellos, S.; Bratschitsch, R. Photoluminescence Emission and Raman Response of Monolayer MoS_2, MoSe_2, and WSe_2. *Opt. Express* **2013**, *21* (4), 4908. https://doi.org/10.1364/OE.21.004908.

(2) Quereda, J.; Ghiasi, T. S.; Van Der Wal, C. H.; Van Wees, B. J. Semiconductor Channel-Mediated Photodoping in h-BN Encapsulated Monolayer MoSe2 Phototransistors. *2D Mater.* **2019**, *6* (2). https://doi.org/10.1088/2053-1583/ab0c2d.

(3) Chernikov, A.; Van Der Zande, A. M.; Hill, H. M.; Rigosi, A. F.; Velauthapillai, A.; Hone, J.; Heinz, T. F. Electrical Tuning of Exciton Binding Energies in Monolayer WS2. *Phys. Rev. Lett.* **2015**, *115* (12), 1–6. https://doi.org/10.1103/PhysRevLett.115.126802.

(4) Haug, H.; Koch, S. W. *Quantum Theory of the Optical and Electronic Properties of Semiconductors*; World Scientific Publishing Company, 2009.

(5) Larentis, S.; Movva, H. C. P.; Fallahazad, B.; Kim, K.; Behroozi, A.; Taniguchi, T.; Watanabe, K.; Banerjee, S. K.; Tutuc, E. Large Effective Mass and Interaction-Enhanced Zeeman Splitting of K -Valley Electrons in MoSe2. *Phys. Rev. B* **2018**, *97* (20), 1–5. https://doi.org/10.1103/PhysRevB.97.201407.
